# Translation and content validity of the Dutch Impact of Vision Impairment questionnaire assessed by Three-Step Test-Interviewing

**DOI:** 10.1186/s41687-020-00272-z

**Published:** 2021-01-05

**Authors:** T. P. Rausch – Koster, A. J. van der Ham, C. B. Terwee, F. D. Verbraak, G. H. M. B. van Rens, R. M. A. van Nispen

**Affiliations:** 1 Amsterdam UMC, Vrije Universiteit Amsterdam, Department of Ophthalmology, Amsterdam Public Health Research Institute, De Boelelaan, 1117 Amsterdam, The Netherlands; 2 Bergman Clinics, Department of Ophtalmology, Amsterdam, The Netherlands; 3 Department of Epidemiology and Biostatistics, The EMGO Institute for Health and Care Research, VU University Medical Center, Amsterdam, The Netherlands; 4 Department of Ophthalmology, Elkerliek Hospital, Wesselmanlaan 25, 5707 HA Helmond, The Netherlands

**Keywords:** Exudative retinal disease, Vision impairment, Health related quality of life, Three-step test-interview, Patient reported outcome measure

## Abstract

**Background:**

Patients suffering from exsudative retinal diseases may experience severe central vision loss and this might have impact on their daily activities and quality of life. To measure the disabilities these patients may have, the use of the Impact of Vision Impairment Profile questionnaire is recommended. The aim of this study was to translate the original English 28-item Impact of Vision Impairment Profile (IVI) into the Dutch language and evaluate its comprehensibility, comprehensiveness and relevance as evidence of content validity. The translation process was performed using standardized methods. Content validity was assessed by cognitive debriefing using a Three-Step Test-Interview (TSTI) method for participants diagnosed with exudative retinal diseases. Step 1 and 2 focused on assessment of comprehensibility of items, step 3 on comprehensiveness and relevance. Audio-recorded qualitative data was analyzed using Atlas.ti. Data regarding comprehensibility problems was further categorized into item-specific problems and general problems.

**Results:**

Few minor discrepancies in wording were found after translation. After conducting 12 cognitive interviews, data saturation was reached. All participants reported comprehensibility problems resulting from specific items, these were; sentence structure, vocabulary and formulation, influence of conditions or composite items and influence of comorbid disorders. Several general comprehensibility problems resulting from instructions or response categories were detected. The main general comprehensibility problem resulted from the layout of the Dutch-IVI. Most participants considered the included items as relevant and indicated that they covered the problems that occur due to vision impairment.

**Conclusions:**

Minor problems in the Dutch translation were detected and adjusted. The layout and instructions of the Dutch-IVI resulted in some comprehensibility problems. The Dutch-IVI appeared to be at risk of being interpreted as a generic patient reported outcome measure, instead of a disease-specific instrument, mainly due to the influence of co-morbidities. Adaptations should improve validity and reliability of the Dutch-IVI, however, cross-cultural comparisons may be at stake.

## Background

Advanced neovascular age related macular degeneration and other retinal exudative diseases, such as diabetic macular edema and cystoid macular edema due to retinal venous occlusion, are the leading causes of legal blindness and low vision in people aged over 50 in developed countries [1, 2]. In contrast to venous occlusion, abovementioned eye diseases often occur in both eyes.

Patients with exudative retinal diseases can experience severe central vision loss within a few days or weeks. Intraocular injections of anti-vascular endothelial growth factor (anti-VEGF) into the eye can be beneficial, i.e. several clinical studies assessing anti-VEGF have shown a significant gain in visual acuity and an increase of vision-related quality of life [3–9]. However, despite beneficial clinical outcomes, patients may find anti-VEGF injections a burden due to the treatment regimen involving the injections and clinical examinations that are often planned (bi-)monthly in the first period of treatment [10]. The treatment focusses on reducing leakages and stabilization of the growth of new blood vessels. Despite these treatments, the loss of central vision affects the daily activities of many of these patients. The impact of visual impairment on quality of life and the psychological burden of vision loss is well known [11–13] .

Clinical measures and performance based measures, such as visual acuity, reading acuity or reading speed and the complications that are directly related to treatment, are routinely registered outcomes in clinical practice [12]. Moreover, Patient Reported Outcome Measures (PROMs), such as quality of life questionnaires, have been developed to evaluate patients’ wellbeing, functioning or disability from their own perspectives, and for this reason are considered meaningful and of direct relevance to patients [12]. Additionally, PROMs are considered a valuable source of information for evaluating health services, establishing treatment effectiveness, informing clinical decision-making and supporting patients in raising their concerns. Moreover, the routine clinical use of PROMs may positively influence doctor-patient communication, shared decision-making and satisfaction with care [12, 14]. The use of PROMs in addition to clinical measures has not gained common ground in ophthalmological practice [12], however, the PROM to evaluate cataract surgeries is being increasingly used.

A working Group, facilitated by the International Consortium for Health Outcomes Measurement (ICHOM), recommended the use of the Impact of Vision Impairment questionnaire (IVI) for patients with exudative retinal diseases [12]. The IVI is a vision-related quality of life questionnaire (PROM) that contains 28 items and was developed to measure how vision impairment impacts on the ability to participate in daily activities.

To develop the IVI, the Centre of Eye Research Australia, conducted patient focus groups to identify all important activities that could result in a restriction of participation. In addition, existing instruments, such as the Bristol Vision-Related Quality of Life (VQOL), the National Eye Institute-Visual Function Questionnaire (NEI-VFQ), the Activities of Daily Vision Scale and the Visual Function (VF)14 questionnaire, were reviewed for relevant content. The first trial version of the IVI consisted of 76 items, of which 69 overlapped with items in the VQOL. Subsequently, the IVI was first developed as a 32-item version and later into a 28-item version and the Brief-IVI has 15 items. All versions contain the 10 core items of the VQOL [12, 15–18], which is known as the Vision Related Quality of Life Core Measure (VCM1). The IVI has been found to be reliable and is responsive to interventions such as anti-VEGF treatment.

The IVI has been translated into eight languages. The brief-IVI has been translated into the Dutch language. However, the full IVI, which is considered useful in creating a more comprehensive item bank for future use in a computer adaptive test, has not been translated yet. A sound translation approach should include qualitative pretesting of questionnaires using cognitive interviews, such as the Three-Step Test-Interview (TSTI) [19–21], to investigate the comprehensibility, relevance and comprehensiveness of the translated items, as evidence of content validity in a new population [22, 23]. The aim of our study was to translate the original full English-language IVI into Dutch and to evaluate its content validity in patients receiving anti-VEGF.

## Methods

This study consisted of two parts: (1) Translation of the original English 28-item IVI into Dutch; (2) Evaluation of its content validity through cognitive debriefing, using the TSTI method.

### Ethical considerations

The study protocol was approved by the Medical Ethics Committee (METC) of Amsterdam University Medical Centers and conducted according to the principles of the Declaration of Helsinki. The METC declared that the protocol did not fall under the scope of the Medical Research Involving Human Subjects Act, that is part of the Dutch law.

### Translation

Before the 28-item IVI was translated, we received access to the Dutch version of the Brief-IVI, consisting of 15 items which were translated in 2017 by a certified translation service into Dutch using adequate forward-backward procedures. Since we were interested in the full version for future studies, additional translation was needed for 13 of the items. This translation process was performed using standardized state-of-the-art translation methods recommended by the Patient-Reported Outcome Consortium [22, 24, 25].

Two independent, forward translations were undertaken by Dutch native speakers. Both translators were familiar with vision-related quality of life questionnaires, and both had a good command of the English language. As quality of life is a subjective construct, the aim of the translations was to render the conceptual meanings of the items. The two forward translations (T1 and T2) were combined into one version (T12) by the translation coordinator. In case of any discrepancies, the two translators and the translation coordinator discussed and compared the two translations in order to produce a reconciled version. Subsequently, the reconciled version (T12) was back translated by two native English translators working independently (TB1 and TB2) from each other and who were both familiar with ophthalmology and PROMs. Both translators were masked from the original items of the IVI. The two back translations were combined into one version (TB12) by the translation coordinator and one of the translators. A few discrepancies in the wording were uncovered between these two translations and were subsequently discussed, resolved and refined by consensus. The back translation was reviewed against the original version by the translation coordinator and a forward translator, and subsequently adjustments in the wording were agreed upon. The Dutch 28-item version was further used as the pre-test version in the semi-structured cognitive debriefing interviews.

### Cognitive debriefing

#### Setting and participants

The TSTI study was carried out at the Department of Ophthalmology, the Amsterdam University Medical Centers and Bergman Clinics eye hospital, Lelystad, in the Netherlands. Patients were eligible to participate if they met the following criteria: 18 years or older, able to read, speak and understand Dutch, no cognitive disability (measured with the 6-item version of the Mini Mental State Examination administered by telephone) and currently receiving anti-VEGF treatment for any exudative retinal disease that can potentially cause visual impairment (i.e. Wet-age related macular degeneration, diabetic macular edema or secondary cystoid macular edema due to retinal venous occlusion). Patients receiving anti-VEGF injections for exudative retinal diseases, the target population of this study, were informed about this study through written study information. Potential eligible participants received the study information from their ophthalmologist and they were asked if they agreed to be contacted by telephone by the researchers. Potential participants were contacted by telephone to ask if they were willing to participate, after which the assessment whether the patients met the inclusion criteria was done. In order to explore a broad range of views on the items, selective sampling was used for the degree of visual complaints indicated by the patient, their age, gender and level of education, to ensure sufficient variability within our group of participants. We focused during the inclusion phase by telephone, on these patient characteristics. We included 12 patients. All patients gave their written, informed consent and all the interviews were conducted at the patients’ homes.

#### The three-step test-interview

The TSTI method can be used to identify problems with self-completion questionnaires by producing observational data of the response behavior of the participants. The first step of the TSTI consists of collecting respondent-driven information about patients’ behavior during the completion of the questionnaire (observation). The aim is to evaluate if patients understand the items, response options, and instructions in the questionnaire as intended (comprehensibility). Because much of this behavior consists of thinking, and is therefore hidden for the observer, the think-aloud-technique is used. Participants were asked to verbalize everything they were thinking about when answering the questions. The second step aimed to fill in the gaps in observational data gathered in step one by using probes such as ‘*I heard you sigh at question [ … ] can you indicate what you were thinking at that moment?’* or *‘You seemed hesitant at question [ … ], can you indicate what exactly caused you to pause?’*. The third step focused on the comprehensiveness of the questionnaire and relevance of the items by eliciting experiences and opinions as in a “regular” cognitive interview, e.g. ‘*Do you think that the questions in this questionnaire cover the problems that you might experience due to your eyesight?’, ‘Do you think there are questions or topics relevant to patients with vision impairment that are not included in the questionnaire?’* or *‘Do you think there are questions in the questionnaire that are not relevant to patients with vision impairment?’* and *‘Were there any questions that you found unpleasant to answer?’*. All interviews were administered using a semi-structured interview guide based on the three facets of content validity; comprehensibility, comprehensiveness and relevance [23].

#### Data analysis

All interviews were audio-recorded and then transcribed in order to summarize the results. Analysis was performed using Atlas.ti (Version 7.5.16). Firstly, problems (first and second phase of TSTI) and comments (third phase TSTI) were structured. As patients’ comprehensibility of items is determined by a series of factors, we divided the item-specific problems into several categories, for example, ambiguity of items, items containing double-barreled questions as well as complex sentence structure or length of the items, specificity of item-wording, vocabulary or the use of jargon. In addition, the comprehensibility of a questionnaire is also determined by non-item-specific factors, for example the layout or the structure of the questionnaire, or written instructions. These general problems were categorized as well, as were all problems and comments that arose during the cognitive interviews to create a structured overview. Transcripts were coded by one researcher against the three areas of content validity; comprehensibility, comprehensiveness and relevance. The comprehensibility area was divided into two subthemes; item-specific comprehensibility problems and general comprehensibility problems.

## Results

### Translation

The two forward translations showed no major differences, only a few minor variations in wording = (7 items). These differences were discussed and solved, and a consensus was reached. In the translations of item 2 ‘*Taking part in recreational activities such as bowling, walking or golf*’ there was a difference in the translation of the specific activities that were mentioned in the question. Since recreational activities are culturally dependent and, additionally, age dependent, we included a specific question in the interview (Step 3 of TSTI) were we asked participants for suggestions for this item.

Backward translations showed only a few minor wording discrepancies. For example, item 6 ‘*paying attention or devoting attention to your appearance*’. Additionally, there were differences in the use of the simple present and present perfect for items 10 and 12. The differences were solved in discussion and refined by consensus.

### Cognitive debriefing interviews

Initially, we targeted to include 12 patients in this study. Since patient inclusion and the interviewing of participants was performed by the same researcher, it was possible to properly judge when data saturation was reached. Saturation was defined as ‘no new problems regarding comprehensibility, comprehensiveness and relevance of the Dutch IVI are mentioned. After 12 patients were interviewed, data saturation was reached. The group consisted of 8 women and 4 men, aged between 67 and 85 years, with a mean age of 75.2 years (SD 6.0). Nine participants indicated that their binocular visual complaints were ‘few’ to ‘moderate’. Three participants experienced binocular visual complaints which they described as ‘many’ to ‘severe’. In nine participants the level of education resulted in a median of 10 years [0, 15]. Of three participants their level of education was unknown. The study sample represents the target population.

#### Comprehensibility problems

Four item-specific, comprehensibility problem categories arose; namely, those resulting from sentence structure (1), vocabulary and formulation (2), influence of conditions or composite items (3) influence of comorbid disorders (4). Furthermore, three general response problems resulted from: not understanding when to choose the response categories ‘never’ and ‘n/a’ (5), misinterpreting the instructions (6), and, not understanding the layout or structure of the questionnaire with regards to ‘presentence’ (7). See Table 1 for an overview of comprehensibility problems, citations and potential solutions.

**Table 1:** Comprehensibility problems of the Impact of Vision Impairment (IVI) (*n* = 12) and possible solutions

Subcategory	Problem description	Item-formulation	Quotations	Possible solutions for the Dutch version IVI
**Item specific problems– Sentence structure**	Comprehensibility problems resulting from (complex) sentence structure	(Item 12) In general, how much has your eyesight interfered with travelling or using transport? (bus and train)	“Only buses and trains were specifically mentioned, so I just thought of these options in my response”	Changing *and* to *or (*Bus *or* train) to clarify these are examples and not a ‘checklist’.
**Item specific problems– vocabulary and formulation**	Comprehensibility problems resulting from the use of vocabulary, unclear formulation or lack of specified formulation	(Item 3) Shopping? Finding what you want and paying for it?	(R5) “Shopping … I don’t go shopping” (Step 1 (TSTI) (R5) “I did not interpret the question widely, for example including shopping for *groceries,* I only thought about buying clothes, that is what the word *shopping* means to me” (Step 2 TSTI)	Adding ‘for example groceries’
		(Item 15) Getting information that you need?	(R5) “What kind of information is meant here? Information concerning my eyes? I do not understand the meaning of the question” (R6) “Do they mean reading information about medicines here?” (R7) “What is meant with information here? Perhaps what I have to read?” (R9) “Information that I have to read? Ah.. Yes I think it is information that I read” (R8) “Is that information that I get using my iPad?”	Clarify what kind of information (*read)*.
		(item 19) In the past month, how often has your eyesight stopped you from doing the things you want to do?	(R7) “How often … does that means I have to fill in a number”	Changing the formulation of an item to fit with response category
**Item specific problems– influence of conditions or composite items**	Comprehensibility problems resulting from different conditions while performing a task that can influence the response or any problems resulting from composite items	(Item 2) Taking part in recreational activities such as …	(R8) “This would only apply to me when it gets dark. I can’t answer this question”	Adding conditions in item formulation, for example *under normal lighting conditions*
		(Item 5) Recognizing or meeting people?	(R3) “Actually, I have no problems at all meeting people, but recognizing people is very difficult for me” (R12) “Well … that depends on the lighting conditions”	Split item; recognizing and meeting people
		(Item 12) In general, how much has your eyesight interfered with travelling or using transport? (bus and train)	(R9) “Well, I never use public transport and we do not travel anymore … I think that is easy to answer … ehm … well … actually, we do travel by car and fortunately, I don’t have any problems driving a car.” (R10) “Yes.. that is different: I know which bus I have to take and where to get off. I have often travelled by train, but now there are a lot of new stations which makes it more difficult for me to travel. But I still do it.” (R12) “I have no problems taking the bus, but I have only taken the train once.”	Split item; travelling could be interpreted as driving a car; separate item
**Item specific problems– influence of comorbid disorders**	Comprehensibility problems resulting from items not necessarily targeting vision problems (answers based on interpretation of the question for comorbid disorders)	(Item 3) Shopping? (finding what you want and paying for it)		Items 3, 4, 7, 17, 18: include *eyesight* in item formulation Items 10, 11, 19 (*and 3, 4, 7, 17, 18)*: clarify ‘n/a’ response category for example *don’t do this for other reasons than because of my eyesight (general comprehensibility)*
		(Item 4) Visiting friends or family?		
		(Item 7) Opening packaging? (For example, around food, medicines)	(R1) “I find it difficult, but that has to do with my hands, not because of my eyesight.” (R3) “This is difficult, but has nothing to do with my eyesight, but with how things are packaged sometimes” (R6) “Actually, this does not apply to my eyes, but I have trouble with it, so I chose the answer ‘not applicable’.”	
		(Item 10) How much has your eyesight interfered with getting about outdoors? (on the pavement or crossing the street)		
		(Item 11) In the past month, how often has your eyesight made you go carefully to avoid falling or tripping?		
		(Item 17) Spilling or breaking things?	(R12) “Yes, sometimes, which has to do with my [comorbid disorder] and not because of my eyes”	
		(Item 18) Your general safety when out of your home?		
		(item 19) In the past month, how often has your eyesight stopped you doing the things you want to do?	(R12) “Yes, sometimes, which has to do with my [comorbid disorder] and not because of my eyes”	
**Problems response category**	Responding problems resulting from not understanding when to choose response categories ‘never’ and ‘n/a’	n / a		Include examples in instructions in which the situation ‘never’ and ‘n/a’ response category is suitable. Clarify ‘n/a’ response category for example *I don’t do this for reasons other than my eyesight.*
**Problems with instructions**	Responding problems resulting from misinterpreting or forgetting the instructions	n / a		Repeatedly include the instructions in the questionnaire, and where possible, included them in item-formulation (shortened version or remark)
**Problems with instructions**	Responding problems resulting from not understanding the layout or structure of the questionnaire regarding the ‘presentence’	n / a	“Despite the sentences being longer, the readability and understandability of the questions will improve”	Include ‘presentence’ in all item formulations.

#### Comprehensiveness

One participant indicated not to have experienced many problems in daily life due to the eye condition, making it difficult to judge if the questionnaire was complete. Another participant thought that more questions needed to be included in the questionnaire, but at the time could not come up with any missing topics. Ten participants indicated that they found the items included covered the possible problems that occur due to vision impairment. However, one participant mentioned missing items in the questionnaire with regards to seeing colors and the ability to estimate depth. Furthermore, one participant suggested adding a question about reading cooking instructions or other instructions and manuals. Adding an open item for any extra information about daily problems or other issues, was also suggested as a way to improving comprehensiveness.

#### Relevance

In step 3 of the TSTI (‘regular interview’) all the participants found the included items relevant to patients with visual impairment. However, two participants mentioned they thought that item 21 ‘*Have you felt embarrassed because of your eyesight?*’ was a strange question. On the other hand, they could imagine that some patients may have this feeling in certain situations.

Furthermore, item 7 ‘*Opening packaging? (For example, around food, medicines)’* turned out to be an item of which seven participants indicated they experienced problems but not due to their eyesight.

Item 2 *‘Taking part in recreational activities such as bowling, walking and golf’* we conceptually translated into *‘cycling, walking or other activities’,* most participants found these examples more relevant to Dutch culture. Two participants suggested mentioning an example that was not a physical activity, such as doing a jigsaw puzzle. Adaptations to the Dutch-IVI are presented in bold in Table 2.

**Table 2:** Adaptation to the Dutch Impact of Vision Impairment Profile

Original IVI	Dutch-IVI (final version)
	General changes: ▪ Inclusion of the ‘**presentence**’ in several items (see item 1 and 16 for example) ▪ Frequently repeating the instructions ▪ Clarifying n/a response option
INSTRUCTIONS Please read each question carefully and circle the answer that BEST applies to you. Put one circle on each row. If you use GLASSES, CONTACT LENSES OR MAGNIFIERS for some activities please answer according to how you can see when using them.	INSTRUCTIONS Please read each question carefully and circle the answer that BEST applies to you. Put one circle in each row. If you use GLASSES, CONTACT LENSES OR MAGNIFIERS for some activities please give your answer according to how you see when using them. **Choose the answer ‘never’ if you can perform the task without being hindered by your vision. Choose ‘not applicable’ if you do not perform the task for reasons other than your vision.**
PRESENTENCE: In the PAST MONTH, how much has YOUR EYESIGHT INTERFERED with the following activities:	
1. Your ability to see and enjoy T.V?	1. **In the past month, how much has your eyesight interfered with** your ability to see and enjoy T.V.?
2. Taking part in recreational activities such as bowling, walking or golf?	2. **[presentence]** taking part in recreational activities such **as cycling, walking or other activities**?
3. Shopping? (finding what you want and paying for it)	3. **[presentence]** shopping (**for example for groceries)** (finding what you want and paying for it)?
4. Visiting friends or family?	4. **[presentence]** visiting friends or family?
5. Recognizing or meeting people?	5a. **[presentence] meeting** people?
	5b. **[presentence] recognizing** people?
6. Generally looking after your appearance? (face, hair, clothing etc.)	6. **[presentence]** generally looking after your appearance? (face, hair, clothing etc.)
7. Opening packaging? (for example, around food, medicines)	7. **[presentence]** opening packaging? (for example around food, medicines)
	**SHORT REPEAT INSTRUCTIONS: Please answer the following questions about YOUR eyesight with GLASSES, CONTACT LENSES or MAGNIFIERS, if you use them.**
8. Reading labels or instructions on medicines?	8. **[presentence]** reading labels or instructions? (for example, on medicines)
9. Operating household appliances and the telephone?	9a. **[presentence]** operating **household appliances**? (**for example, the washing machine, oven, microwave or thermostat**)
	9b. **[presentence]** operating your **(mobile) phone**?
10. How much has your eyesight interfered with getting about outdoors? (on the pavement or crossing the street)	No changes to item
11. In the past month, how often has your eyesight made you go carefully to avoid falling or tripping?	No changes to item
12. In general, how much has your eyesight interfered with travelling or using transport? (bus & train)	12. In general, how much has your eyesight interfered with travelling or using transport? (bus **or** train)
13. Going down steps, stairs, or curbs?	13. **[presentence]** going down step, stairs, or curbs?
	**SHORT REPEAT INSTRUCTIONS: Please answer the following questions about YOUR eyesight with GLASSES, CONTACT LENSES or MAGNIFIERS, if you use them.**
PRESENTENCE: In the PAST MONTH, how much has YOUR EYESIGHT INTERFERED with the following activities	
14. Reading ordinary size print? (for example, newspapers)	14. **[presentence]** reading ordinary size print? (for example, newspapers)
15. Getting information that you need?	15. **[presentence]** getting information that you need?
SHORT REPEAT INSTRUCTIONS: Please answer about YOUR eyesight with GLASSES, CONTACT LENSES or MAGNIFIERS, if you use them.	Same instructions added
PRESENTENCE: In the PAST MONTH, how often has YOUR EYESIGHT MADE YOU CONCERNED OR WORRIED about the following:	
16. Your general safety at home?	16. **In the past month, how much has your eyesight made you concerned or worried about** your general safety at home?
17. Spilling or breaking things?	17. **[presentence]** spilling or breaking things?
18. Your general safety when out of your home?	18. **[presentence]** your general safety when out of your home?
19. In the past month, how often has your eyesight stopped you doing the things you want to do?	No changes to item
20. In the past month, how often have you needed help from other people because of your eyesight?	No changes to item
SHORT REPEAT INSTUCTIONS: Please answer about YOUR eyesight with GLASSES, CONTACT LENSES or MAGNIFIERS, if you use them.	**DELETE instructions because of domain: emotional well being**
INSTRUCTIONS: Think about how YOUR eyesight has made you FEEL in the PAST MONTH.	**Same instructions added**
21. Have you felt embarrassed because of your eyesight?	No changes to item
22. Have you felt frustrated or annoyed because of your eyesight?	No changes to item
23. Have you felt lonely or isolated because of your eyesight?	No changes to item
24. Have you felt sad or low because of your eyesight?	No changes to item
25. In the past month, how often have you worried about your eyesight getting worse?	No changes to item
26. In the past month how often has your eyesight made you concerned or worried about coping with everyday life?	No changes to item
27. Have you felt like a nuisance or a burden because of your eyesight?	No changes to item
28. In the past month, how much has your eyesight interfered with your life in general?	No changes to item

## Discussion

In this study the translation of the 28-item IVI into the Dutch language was described and its content validity was investigated by evaluating the comprehensibility, comprehensiveness and relevance of the items. The TSTI technique, including the think-aloud-method, enabled identification of problems that are usually invisible to researchers when using self-report questionnaires.

In our study all of the participants encountered comprehensibility problems with several items or indicated general comprehensibility problems regarding the response category and the questionnaire’s instructions. Most of the item-specific problems were minor issues regarding translation that could be solved with small changes to the wording or specifying the phrasing and clarifying the item context or conditions. Several items contained composite questions, and as expected, these caused problems as they contain the risk of being understood and answered ambiguously [26]. Comprehensibility is probably influenced by the level of education of the respondents. We believe that the variation in level of education was sufficient among our respondents, however in 25% of our study population the level of education was unknown. The key findings of this study are the general comprehensibility problems into which the TSTI provided insight. Paying attention to these issues is extremely important when developing questionnaires. In our study we found eight items in the IVI that are at risk of being interpreted and answered based on comorbid disorders, which hampers the validity of the questionnaire. We recommend to provide a clear instruction on how to complete the questionnaire, and, to repeat this, where possible, throughout the questionnaire. For specific items, it’s worth considering whether a shortened version of the instructions should be included with the item itself. This could prevent disease-specific instruments being answered as general instruments by participants. In our study, the participants who encountered problems regarding the ‘presentence’, thought the items would have been clearer if the ‘presentence’ was included in each item. However, there is an argument as to whether this will improve the readability, and in turn comprehensibility, for all participants. We considered that most of the comprehensibility problems we identified with TSTI needed to be adjusted in order to finalize the Dutch translation. We decided not to adjust item 2, 15 and 19 as the frequencies in which these item-specific problems occurred were low. Despite the fact that item 9 ‘*Operating household appliances and the telephone’* did not cause any problems during our interviews, we decided to split this item because of the composite structure of the question. Since patients increasingly use mobile phones or smartphones; we believe using the telephone differs greatly from the operation of household appliances. These adaptations should improve the validity and reliability of the Dutch-IVI, however, cross-cultural comparisons may be at stake.

PROMs have been proven to contribute to doctor-patient communication [14]. In our study, one participant suggested adding an open question to the IVI. Even though most of our participants mentioned the questions included in the IVI cover all the daily problems that patients with visual impairment may experience, it is worth considering allowing patients the opportunity to write openly about their problems, thus making the questionnaire even more patient-specific. Consequently, to ensure that patients are motivated to complete PROMs, it is important that clinicians use their answers in communication with the patient, for example in shared-decision-making processes [14]. In addition to the participants’ view of the comprehensiveness of the IVI, we find that some domains of vision-related quality of life may be under-represented or even not represented at all, such as ‘driving/ transport’ and ‘bodily symptoms /functions’, two out of the nine domains the working group (ICHOM) felt were most important to patients with macular degeneration [12]. Apart from item 12, which only focuses on transport, there is no item regarding driving a car included. Moreover, adding items that describe specific conditions, for example low lighting, may improve the sensitivity of the IVI and possibly make this PROM more valuable for patients who experience fewer eye complaints due to retinal diseases, as several studies confirmed that patients have more difficulty performing tasks under low illumination, even if they are free of ocular diseases [27].

Interviewing individuals from the target population about their perception of the questionnaire using the TSTI method showed that the IVI provides an adequate reflection of the construct it is intended to measure, namely vision-related quality of life. All participants found that the items in the IVI were relevant to the problems they might have due to vision impairment. When selecting items for the development of health-related quality of life questionnaires, evaluating patients’ perspectives on the content validity of a questionnaire is considered to be essential, in addition to its assessment by experts in the field. The last phase of TSTI especially provided the opportunity to evaluate the participants’ views on comprehensiveness, and also on the relevance of the items [23, 24, 26].

## Conclusions

Due to the interviews using the TSTI method, minor problems in the wording and formulation of the Dutch translation of the items were addressed. Furthermore, this study clearly showed several general problems regarding the layout or structure and instructions of the questionnaire. This disease-specific PROM appeared to be at risk of being interpreted as a generic PROM by participants. We recommend making patients aware of the importance of giving their response to the questions based on their eye-sight and, where possible, including this in item formulations. These recommendations can be broadened to other fields where disease-specific quality of life questionnaire are being translated or developed. The cognitive interviews provide qualitative evidence of the relevance, comprehensibility and comprehensiveness of any translated instrument and additionally, problems due to structure or layout. In our study, the TSTI method helped detect and adjust the majority of possible participants’ comprehensibility problems. As a result the adaptations we made should contribute to content validity. The comprehensiveness of the Dutch IVI is assumed to be good, however adding more items may improve its sensitivity in general and its applicability to patients who experience fewer visual complaints.

## Data Availability

The datasets used and/or analyzed during the current study are available from the corresponding author on reasonable request.
